# Retraction Note: Human induced pluripotent stem cell-derived platelets loaded with lapatinib effectively target HER2+ breast cancer metastasis to the brain

**DOI:** 10.1038/s41598-024-56291-z

**Published:** 2024-03-12

**Authors:** Arunoday Bhan, Khairul Ansari, Mike Y. Chen, Rahul Jandial

**Affiliations:** 1 Division of Neurosurgery, Beckman Research Institute, City of Hope Medical Center, 1500 E. Duarte Rd, Duarte, CA 91010 USA; 2 Celcuity LLC, Minneapolis, MN 55446 USA

Retraction of: *Scientific Reports* 10.1038/s41598-021-96351-2, published online 15 October 2021

The Editors are retracting this Article.

This follows an investigation by the City of Hope Medical Centre that found discrepancies in the data. Specifically:

- In Supplementary Fig. 1B, the fluorescence microscopy image of a culture of hiPSC line DF-19-9-7T was obtained from a derivative of hiPSC line 1157.2, specifically targeted using CRISPR to delete the gene ATRX.
- In Supplementary Fig. 1E, the electron microscopy image of a megakaryocyte on day 6 of maturation was not obtained directly by differentiation of commercially available hiPSC line DF-19-9-7T as described, but instead from an immortalized megakaryocyte cell line (four days after doxycycline-withdrawal induction of differentiation), previously derived from a hiPSC line.
- In Supplementary Fig. 1C, the karyotype image from a cell from the hiPSC line DF-19-9-7T was obtained from hiPSC line 1156.

Additional, the investigation found that these data and the electron microscopy image presented in Fig. 2D were not owned by the Authors. The Editors no longer have confidence in the reliability of the data reported.

Khairul Ansari, Mike Y. Chen and Rahul Jandial agree with the retraction and its wording. Arunoday Bhan did not respond to the correspondence from the Editors about the retraction.

